# The correlation between temperament and adherence for HIV and TB patients

**DOI:** 10.1186/1471-2334-13-S1-P107

**Published:** 2013-12-16

**Authors:** Daniela-Ica Biriş, Teodora Moisil, Elena-Voichița Lăzureanu

**Affiliations:** 1 Dr. Victor Babeş Clinical Hospital of Infectious Diseases and Pneumology, Timişoara, Romania; 2 University of Medicine and Pharmacy “Victor Babeş” Timişoara, Romȃnia

## Background

The patient's temperament plays an important role in the success or failure of any treatment. The temperament is the dynamic-energy dimension of the personality which is mostly seen in the demeanor. On the other hand the adherence is the correlation between the patient's behavior and the medical recommendation adapted to the patient needs. Non-adherence to treatment and the factor that influences it is a continuous concern and for this reason we launched the hypothesis: “Does the behavior have an influence on the adherence of patients suffering from HIV and TB infection?”

## Methods

The comparative study was performed on a sample of 60 patients: 30 patients with HIV (15 new cases and 15 experienced) and 30 patients with TB (15 new cases and 15 with recurrence). We applied three questionnaires: the Heymans temperamental questionnaire, the questionnaire for evaluating the adherence for antiretroviral treatment and the questionnaire for evaluating the adherence for the antituberculous treatment.

## Results

The results show a significant non-adherence of the patients with a phlegmatic, choleric or passionate temperament, these results being confirmed both in the HIV and TB patients.

## Conclusion

Being aware of the temperament of the HIV and TB patients can eliminate one of the unknown barriers of non-adherence by a cautious observation. The patients with a phlegmatic, choleric or passionate temperament are more predisposed to non-adherence. From a psychological point of view, we can agree that the temperament has an influence over the adherence.

